# Cardiovascular risk factors and quality of life among stroke survivors in Korea from 2013 to 2018: a cross-sectional cohort study

**DOI:** 10.1186/s12955-022-02008-7

**Published:** 2022-06-27

**Authors:** Hyejin Jung

**Affiliations:** Department of Meridian & Acupoint, College of Korean Medicine, Kyung Hee University, 26 Kyungheedae-ro, Dongdaemun-gu, Seoul, 02447 South Korea

**Keywords:** Cardiovascular health, Quality of life, Ischemic heart disease, Stroke

## Abstract

**Background:**

Although the cardiovascular health and quality of life (QoL) of stroke survivors have been previously studied, no study has investigated the correlation between cardiovascular health and QoL. This study aimed to investigate whether there would be a difference in the quality of life (QoL) in this population depending on the degree of cardiovascular health.

**Methods:**

Overall, 577 people aged > 40 years who participated in the Korea National Health and Nutrition Examination Survey from 2013 to 2018 were included and were divided into three groups according to the survey period (2013–2014, *n* = 145; 2015–2016, *n* = 198; and 2017–2018, *n* = 234). Participants were further divided into the following groups based on their cardiovascular health score, as defined by the American Heart Association: poor, intermediate, and ideal groups. We examined how the health-related QoL score was expressed through the five-dimensional European Quality of Life Questionnaire (EQ-5D-3L).

**Results:**

The ideal (cardiovascular health scores 11–14) and intermediate (cardiovascular health scores 8–10) groups had the lowest (7.72–8.14%) and highest (46.39–57.70%) number of participants, respectively. The total EQ-5D index score was highest in the ideal group, followed by the intermediate and poor groups across all three periods (2013–2014, *p* = 0.0015; 2015–2016, *p* = 0.0040; 2017–2018, *p* < 0.0001). The dimension-specific analysis revealed that, Findings showed that stroke survivors' mobility significantly varied by cardiovascular health scores (*p* = 0.0371 in 2015–2016, *p* =0.0486 in 2017–2018), whereas usual activities (*p* = 0.0322) and pain/discomfort (*p* = 0.0420) were significantly different among the three groups in 2015–2016.

**Conclusion:**

QoL in post-stroke survivors, when related to cardiovascular health degree, could be correlated with stroke sequelae.

## Introduction

Over the past few decades, cardiovascular disease (ischemic heart disease, stroke) had been the primary cause of death globally [1]. The overall burden of stroke and related deaths among stroke survivors is dramatically increasing in Korea [2, 3]. Due to an aging population, the burden of stroke is on the increase, hence, it is necessary to understand the characteristics of Koreans with respect to this disease and its related care. Stroke survivors have a higher risk of neurological, neuropsychiatric, vascular, and mortality-related complications than the general population [4, 5]. Stroke survivors are also at risk of recurrent strokes, and secondary strokes are associated with a higher mortality rate and poor functional recovery compared to index strokes [6, 7]. While the early risks of stroke recurrence are highly correlated with the nature of the first stroke, long-term risks are predominantly influenced by the underlying vascular risk factors versus the pattern of the initial stroke [8]. Several vascular risk factors, such as hypertension, diabetes, dyslipidemia, obesity, smoking, unhealthy diet, and physical inactivity are associated with cardiovascular disease [9–11]. It is important to maintain cardiovascular health in stroke survivors while providing appropriate medical treatment to prevent a secondary stroke [12] Studies among stroke patients have shown a lower HRQoL before and after the onset of a stroke compared to the general population [13–15]. Comparison of post-stroke patients and healthy group reveals that cardiovascular disease status was associated with a significantly lower quality of life [16]. In a study on the quality of life of stroke survivors, the EQ-5D index is found to be lower in stroke survivors with hypertension and diabetes mellitus [17].

Insufficient national studies on this topic have been conducted in the Korean stroke survivor population for overall cardiovascular health. Therefore, little is known about HR-QoL according to cardiovascular health in Korean patients after stroke. Tracking the cardiovascular health and HR-QoL of stroke survivors over time is helpful in identifying health disparities, assessing the progress toward broader health care, and establishing public health policies for these patients.

This study investigated the degree of physical health and health-related quality of life by tracking cardiovascular health indicators of stroke survivors using the results of the Korea National Health and Nutrition Examination Survey (KNHANES) to further evaluate this hypothesis.

## Methods

### Study population

We used cross-sectional data from the participants of the KNHANES, a nationwide survey conducted between 2013 and 2018. The KNHANES collected demographic, social, health and nutritional information in the form of health interviews, health screenings, and nutrition questionnaires [18].

The KNHANES utilized households as sampling units through a stratified and multistage cluster probability sampling design based on sex, age, and geographic area using household registries. To ensure an equal probability of being selected, statistical weights were assigned to each participant, which allows the results to represent all non-institutionalized Korean individuals [18]. All surveys were conducted after obtaining informed consent from the participants. The KNHANES is reviewed and approved by the Research Ethics Review Board of the Korea Centers for Disease Control and Prevention (KCDC) annually. This study was conducted in accordance with ethical standards, as laid down in the 1964 Declaration of Helsinki and its later amendments or comparable ethical standards. Using data provided online for research, this study conducted a secondary analysis. The number of subjects who undertook the health surveys, checkups, and nutrition surveys from 2013 to 2018 was 39,642. There were 22,763 people aged > 40 years, of which 754 were stroke survivors who were diagnosed with a stroke by a doctor. Patients under 40 years of age were excluded from the research because they had a distinct mechanism for their stroke [19].

Finally, this study included 145 patients in 2013–2014, 198 patients in 2015–2016, and 234 patients in 2017–2018, excluding those with missing information on the 7 Cardiovascular Health Metrics and five-dimensional European Quality of Life Questionnaire (EQ-5D) and pregnant or lactating women.

### 7 Cardiovascular health metrics score

The cardiovascular health status of stroke survivors was quantified using the 7 Cardiovascular Health Metrics as defined by the AHA [20]. The metrics include seven modifiable risk factors consisting of current smoking status, body mass index (BMI), physical activity, diet, total cholesterol, blood pressure, and fasting plasma glucose, which were designed to measure health behaviors and biological factors as part of a strategy to prevent cardiovascular disease [20]. It is considered that a health status examination based on these seven metrics could serve as a good indicator of cardiovascular health in stroke survivors. Each metric was scored using 0, 1, and 2 points. Poor health was defined as a score between 0 and 7, intermediate health as a score between 8 and 10, and ideal health as a score between 11 and 14 [21]. The score for each category is based on the AHA guidelines; however, taking differences in race, diet, and survey into account, some modifications were made to suit the Korean population.

Based on the BMI, we created the following subgroups: the ideal group (BMI < 23 kg/m^2^), intermediate group (23 kg/m^2^ ≤ BMI < 25 kg/m^2^), and the poor group (BMI ≥ 25 kg/m^2^) [22]. Physical activity was measured using the International Physical Activity Questionnaire (IPAQ)-short form [23]. According to the IPAQ-short form, the patients were divided in the low, moderate, and high groups according to whether they scored 0, 1, and 2 points, respectively. The patients were further classified into the following groups based on their dietary scores: “non-adherent” (> 2400 mg of sodium intake and low quartiles [Q1–Q3] of the Korean Healthy Eating Index [KHEI] score), “slightly adherent” (either ≤ 2400 mg of sodium intake or the highest quartile [Q4] of the KHEI score), and “highly adherent” (both ≤ 2400 mg of sodium intake and the highest quartile [Q4] of the KHEI score) [24]. The KHEI dietary evaluation index consists of a total of 14 items. Of these, eight items (breakfast, mixed grains intake, total fruits intake, fresh fruits intake, total vegetables intake, vegetables intake excluding Kimchi and picked vegetables intake, meat, fish, eggs and beans intake and milk and milk products intake) are recommended for adequate consumption. Three restricted items (saturated fatty acid energy intake ratio, sodium intake, and sugar and beverage energy intake ratio) are recommended for moderate consumption and three items (carbohydrate energy intake ratio, fat energy intake ratio, and total energy intake) for balanced consumption. These are scored to evaluate the balance of energy intake. The total score of the dietary evaluation index is 100 points, 5 points for multigrain-, fruit-, and vegetable-related items, 10 points for the others, and 10 points for all items in the restricted foods, giving 5 points for balance evaluation [25].

### Quality of life

The HRQoL was measured by the EQ-5D-3L questionnaire, which includes mobility, self-care, daily activities, pain/discomfort, and anxiety/depression. It is structured such that participants must select one of the following answers: “no problem,” “some problems,” and “severe problems.” In this study, “some problems” and “severe problems” were grouped together and referred to as “problem.”

The QoL index (EQ-5D index score) was calculated by using the Time Trade Off method [26].It was calculated as a weighted index value from complete health status classified as 1 to the lowest score classified as − 0.171. EQ-5D in the KNHANES data is the Korean version of EQ-5D, and the Korean weighting of EQ-5D was applied.

### Other variables

To examine the characteristics of a population of patients with stroke, the following variables were investigated: age, sex, marital status, education level, residential area, economic level, economic activity, time after stroke, and current alcohol consumption. Among those diagnosed with stroke by a doctor, the duration of the disease was indicated based on a questionnaire that reported the age of the first diagnosis. The economic status that was represented by equivalent income was calculated as average monthly household income/number of household members and then expressed as a quartile.

### Statistical analysis

For the demographic data of stroke survivors, values are weighted mean ± standard error (SE) or weighted percentage (SE) unless otherwise indicated. The *p*-values were calculated by analysis of variance or the Rao–Scott chi-square test, as appropriate, with *p*-values of < 0.05 being considered statistically significant. Regarding the prevalence of Cardiovascular Health Metrics among participants with a history of stroke, age and sex were adjusted for each category and the *p*-values were calculated by logistic regression analysis adjusted for age and sex. To determine the difference in QoL among the poor, intermediate, and ideal groups according to period, age and sex were corrected using “having a problem” for each of the five EQ-5D items as dependent variables. For the prevalence of “having a problem” in each EQ-5D dimension and EQ-5D index among participants with a history of stroke, *p*-values were analyzed via logistic regression and analysis of covariance adjusted for age and sex. The EQ-5D index scores were calculated as the least squares mean. Differences between years (2013–2014 vs. 2015–2016, 2013–2014 vs. 2017–2018, 2015–2016 vs. 2017–2018) were identified using Tukey–Kramer’s multiple comparison test.

As the data from KNHANES were derived from stratified and multistage cluster probability sampling methods to represent the entire Korean population, general Korean population weighting was also applied in the analyses. The PROC SURVEY procedure was used to apply stratification, primary sampling units, and population weights. Significant levels were set at two-tailed *p*-values < 0.05. All analyses were conducted using SAS version 9.4 (SAS Institute Inc., Cary, NC, USA).

## Results

From 2013 to 2018, 577 out of 22,763 respondents aged > 40 years were identified as stroke survivors. Table 1 shows the demographic characteristics of stroke survivors from 2013–2014 to 2017–2018 in the KNHANES survey. There were significant differences in age and sex among the three groups. The mean age and weighted percentage of female patients in the three groups were as follows: 2013–2014 (age 63.23 years, female 27.0%), 2015–2016 (age 66.43 years, female 39.7%), and 2017–2018 (age 66.95 years, female 43.5%). There was a significant difference in age and weighted percentage of females among the three time periods (age, *p* = 0.0308; sex, *p* = 0.0109). Among stroke survivors, > 95.2% of patients were married, > 87.9% were educated for < 12 years, and > 74.4% lived in urban areas. The patients in the lower quartile of household income accounted for the highest proportion of studied cohort, and the percentage of economic activity of this group was 34.7%–42.9%. The average number of years after stroke was 8.77–9.53 years and the rate of current drinking was 17.1%–18.2%; no significant difference in these characteristics were found among the three periods (Table 1).

**Table 1:** Characteristic of stroke survivors

	2013–2014	2015–2016	2017–2018	*p*-value^*^
N	145	198	234	
Age (years)	63.23 ± 1.12	66.43 ± 0.87	66.95 ± 0.84	0.0308
Sex, female (%)	27.0 (3.56)	39.7 (3.85)	43.5 (3.90)	0.0109
Marital status, married (%)	95.2 (2.31)	97.5 (1.73)	95.7 (1.51)	0.6611
Education level				0.7458
> 12	89.2 (3.30)	90.7 (2.51)	87.9 (2.43)	
≤ 12	10.8 (3.30)	9.3 (2.51)	12.1 (2.43)	
Residential area, urban (%)	74.4 (4.99)	83.3 (3.35)	81.9 (3.11)	0.2640
Household income (%)				0.5503
1Q	40.9 (5.34)	41.0 (4.50)	32.7 (3.58)	
2Q	21.4 (4.13)	20.5 (3.04)	23.1 (3.21)	
3Q	15.3 (3.21)	16.5 (2.80)	23.1 (3.54)	
4Q	22.4 (4.38)	22.0 (3.43)	21.1 (3.20)	
Economic activity, yes (%)	42.9 (4.94)	34.7 (4.27)	35.4 (3.72)	0.3921
Time since stroke onset (years)	8.77 ± 0.57	9.16 ± 0.54	9.53 ± 0.63	0.1536
Current drinking, yes (%)	18.2 (4.08)	18.1 (2.85)	17.1 (3.12)	0.9627

Values are weighted mean ± SE or weighted percentage (SE), unless otherwise indicated

^*^p-value by ANOVA or Rao–Scott chi-square test, as appropriate

The prevalence of the Cardiovascular Health Metrics is shown in Table 2. The prevalence was the highest for those who did not smoke or quit smoking for > 1 year at 83.43–88.39%, and the prevalence for those with intermediate physical activity was at 64.33–66.81%. The prevalence of BMI > 25 kg/m^2^ was 40.78–44.82%, and the health diet score was the highest in the “slightly adherent” group at 48.07–52.54%. The prevalence of total cholesterol level < 200 mg/dL was 75.33–82.09%, and that of blood pressure in the 120–139/80–89 mmHg range was 40.63–42.31%. The prevalence of fasting blood glucose < 100 mg/dL was the highest, ranging from 36.47% to 48.57%. After adjusting for each category of age and sex, the prevalence of total cholesterol in the range of 200–239 mg/dL decreased from 21.75% (95% confidence interval [CI] 14.77–30.84) in 2013–2014 to 19.34% (95% CI 13.87–26.30) in 2015–2016 and to 10.40% in 2017–2018 (95% CI 6.64–15.93). The change was statistically significant (*p* = 0.0226). The proportion of patients with a score between 11 and 14 (ideal group) in 2013–2018 was 7.72%–8.14%, accounting for the least proportion of all stroke survivors, and that of patients with a score between 8 and 10 (intermediate group) was 46.39%–57.70%, showing the highest prevalence (Table 2).

**Table 2:** Prevalence of cardiovascular health metrics among individuals with a history of stroke

	2013–2014		2015–2016		2017–2018		*p*-value^*^
	n	Prevalence (%, 95% CI)	n	Prevalence (%, 95% CI)	n	Prevalence (%, 95% CI)	
*Smoking*							
Current (0)	22	11.10 (6.83–17.54)	35	14.02 (9.21–20.78)	39	14.23 (9.73–20.35)	0.6719
Quit smoking < 12 months (1)	2	0.53 (0.10–2.62)	4	2.19 (1.06–4.44)	3	1.05 (0.24–4.48)	0.1736
Never or quit smoking ≥ 12 months (2)	121	88.39 (81.92–92.75)	159	83.43 (76.59–88.57)	192	84.56 (78.20–89.32)	0.4367
*Physical activity*							
None (0)	39	25.19 (18.01–34.05)	59	28.39 (22.48–35.15)	78	29.10 (22.09–37.26)	0.7821
Intermediate (1)	93	64.33 (54.36–73.20)	127	65.74 (58.34–72.44)	148	66.81 (58.72–74.03)	0.9255
Ideal (2)	13	9.61 (5.46–16.35)	12	5.52 (2.90–10.27)	8	3.73 (1.67–8.13)	0.1239
*Body mass index (kg/m*^*2*^*)*							
≥ 25 (0)	58	44.82 (35.81–54.19)	90	42.14 (34.61–50.05)	94	40.78 (33.88–48.06)	0.7978
23–24.9 (1)	30	16.83 (11.30–24.33)	47	25.61 (18.63–34.11)	66	28.88 (22.37–36.39)	0.0615
< 23 (2)	57	38.13 (29.57–47.49)	61	32.05 (24.73–40.38)	74	30.08 (23.42–37.71)	0.3778
*Healthy diet score*							
Non-adherent (0)	72	45.27 (35.49–55.42)	81	41.68 (33.94–49.85)	81	36.57 (28.90–45.00)	0.3997
Slightly adherent (1)	63	48.07 (38.10–58.19)	101	51.06 (43.26–58.82)	126	52.54 (44.40–60.55)	0.7989
Highly adherent (2)	10	5.15 (2.65–9.79)	16	5.99 (3.47–10.16)	27	9.00 (5.75–13.81)	0.2910
*Total cholesterol (mg/dL)*							
≥ 240 (0)	4	3.02 (0.90–9.58)	9	4.82 (2.22–10.14)	17	7.12 (4.12–12.02)	0.3808
200–239 (1)	31	21.75 (14.77–30.84)	42	19.34 (13.87–26.30)	28	10.40 (6.64–15.93)	0.0226
< 200 (2)	110	75.33 (65.68–82.98)	147	75.48 (67.99–81.70)	189	82.09 (75.33–87.31)	0.2745
*Blood pressure (mmHg)*							
≥ 140/ ≥ 90 (0)	37	24.33 (17.39–32.94)	52	26.15 (18.84–35.06)	66	31.77 (24.51–40.04)	0.3801
120–139/80–89 (1)	60	41.28 (31.96–51.27)	84	42.31 (34.56–50.45)	102	40.63 (33.50–48.17)	0.9540
< 120/ < 80 (2)	48	34.22 (26.08–43.41)	62	31.45 (24.69–39.10)	66	27.47 (20.53–35.69)	0.5160
*Fasting blood glucose*							
≥ 126 (0)	26	18.36 (11.53–27.95)	40	20.44 (14.23–28.45)	66	30.43 (23.28–38.67)	0.0661
100–125 (1)	54	33.02 (24.92–42.27)	71	35.65 (27.33–44.94)	84	32.92 (25.87–40.84)	0.8798
< 100 (2)	65	48.57 (39.21–58.03)	87	43.62 (35.63–51.96)	84	36.47 (28.97–44.68)	0.1532
*Total*							
Poor CV health score (0–7)	51	33.57 (25.22–43.10)	86	44.01 (35.70–52.67)	95	44.67 (36.22–53.43)	0.1778
Intermediate CV health score (8–10)	80	57.70 (48.85–66.08)	97	47.49 (38.75–56.38)	120	46.39 (38.66–54.29)	0.1376
Ideal CV health score (11–14)	14	8.00 (4.21–14.70)	15	7.72 (4.59–12.70)	19	8.14 (4.72–13.71)	0.9898

Adjusted for age and sex

^*^p-value by logistic regression (adjusted for age and sex)

**Fig. 1:**
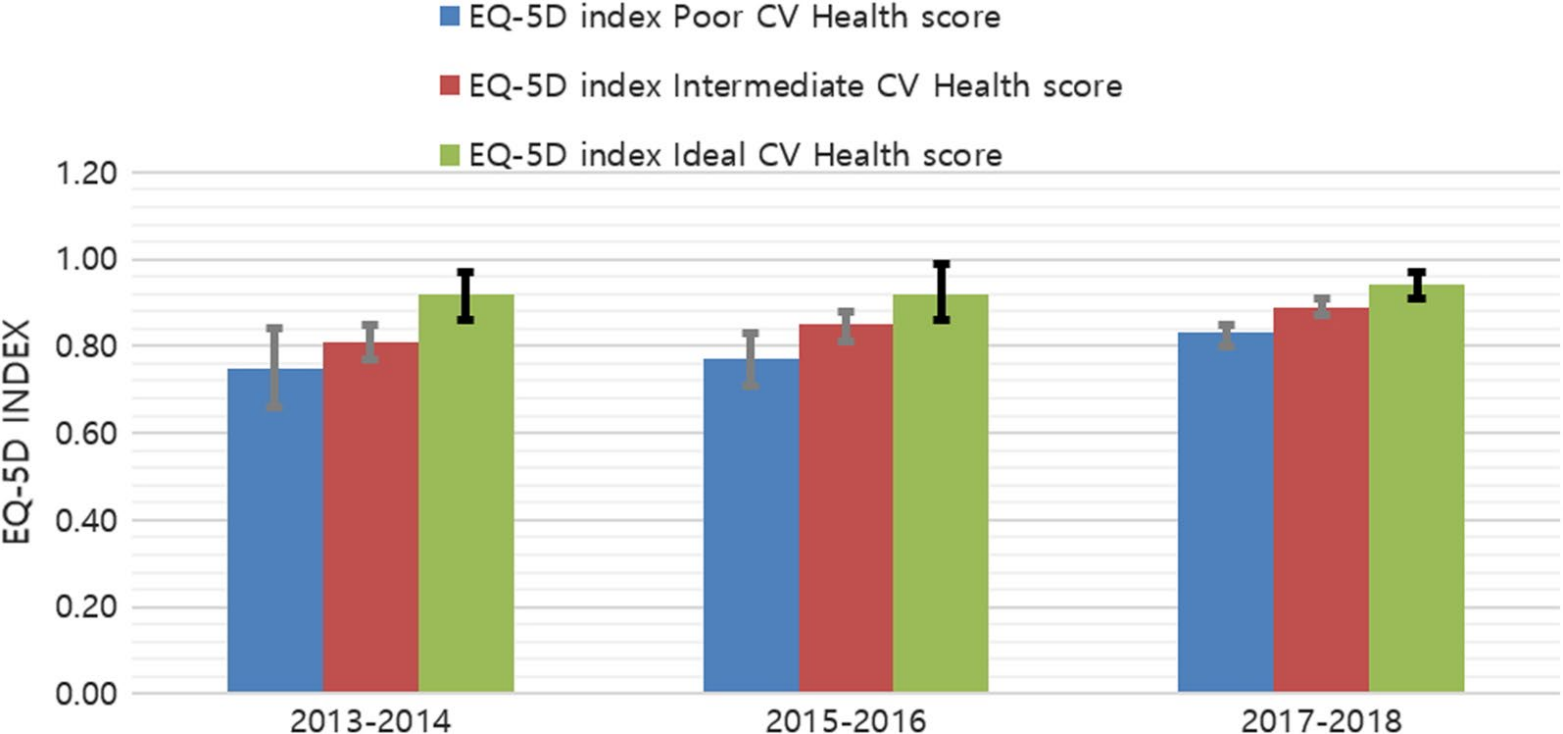
EQ-5D index score among persons with a history of stroke. The EQ-5D index score was calculated as least mean square after sex and age were adjusted, and *p* = 0.0015 in 2013–2014, *p* = 0.0040 in 2015–2016, and *p* < 0.0001 in 2017–2018. EQ-5D, five dimentional European Quality of Life Questionnaire

The total EQ-5D index score was the highest in the ideal group, followed by the intermediate group and finally the poor group across all three periods. After adjusting for sex and age, the poor group had the lowest EQ-5D index score at 0.75 (95% CI 0.66–0.84) in 2013–2014, 0.77 (95% CI 0.71–0.83) in 2015–2016 and 0.83 (95% CI 0.80–0.85) in 2017–2018. The score for the intermediate group was 0.81 (95% CI 0.77–0.85) in 2013–2014, 0.85 (95% CI 0.81–0.88) in 2015–2016, and − 0.89 (95% CI 0.87–0.91) in 2017–2018. Finally, the ideal group had the highest score at 0.92 (95% CI 0.86–0.97) in 2013–2014, 0.92 (95% CI 0.86–0.99) in 2015–2016 and 0.94 (95% CI 0.91–0.97) in 2017–2018. The total EQ-5D index score was significantly different between the poor, intermediate, and ideal cardiovascular health score groups in all time points (2013–2014, *p* = 0.0015; 2015–2016, *p* = 0.0040; 2017–2018, *p* < 0.0001) (Fig. 1).

The dimension-specific analysis revealed no significant difference in all dimensions among the poor, intermediate, and ideal cardiovascular health score groups in 2013–2014. However, in 2015–2016, mobility (*p* = 0.0371), usual activities (*p* = 0.0322), and pain/discomfort (*p* = 0.0420) showed a significant difference among the three groups. In 2017–2018, there was a significant difference in mobility among the groups (*p* = 0.0486) (Table 3).

**Table 3:** Prevalence of having problems in each EQ-5D dimension among persons with a history of stroke (*n* = 577)

		Poor group (0–7)	Intermediate group (8–10)	Ideal group (11–14)	*p*
*2013–2014 (n = 145)*					
Mobility	Prevalence (%, 95% CI)	49.0 (33.3–64.9)	55.7 (43.3–67.4)	29.3 (10.3–60.0)	0.2492
Self-care	Prevalence (%, 95% CI)	35.1 (21.1–52.3)	37.3 (26.7–49.2)	18.2 (5.7–45.2)	0.3755
Usual activities	Prevalence (%, 95% CI)	47.1 (31.5–63.4)	44.1 (32.9–56.0)	18.5 (5.8–45.4)	0.1605
Pain/discomfort	Prevalence (%, 95% CI)	65.4 (48.5–79.1)	52.0 (40.2–63.6)	26.9 (9.1–57.5)	0.1000
Anxiety/depression	Prevalence (%, 95% CI)	38.9 (24.1–56.1)	29.0 (18.2–42.9)	9.7 (2.1–35.5)	0.1179
*2015–2016 (n = 198)*					
Mobility	Prevalence (%, 95% CI)	61.9 (48.4–73.7)	48.3 (37.0–59.7)	24.4 (9.7–49.1)	0.0371
Self-care	Prevalence (%, 95% CI)	35.5 (23.6–49.4)	22.6 (15.1–32.4)	13.9 (4.2–37.3)	0.1202
Usual activities	Prevalence (%, 95% CI)	51.4 (38.4–64.1)	38.5 (28.3–49.8)	16.1 (5.5–38.7)	0.0322
Pain/discomfort	Prevalence (%, 95% CI)	53.5 (40.8–65.8)	45.4 (34.4–56.9)	18.0 (5.9–43.3)	0.0420
Anxiety/depression	Prevalence (%, 95% CI)	35.3 (24.0–48.5)	24.4 (16.9–33.8)	21.9 (7.8–48.2)	0.1914
*2017–2018 (n = 234)*					
Mobility	Prevalence (%, 95% CI)	41.7 (30.2–54.1)	33.3 (22.5–46.3)	10.9 (3.2–31.2)	0.0486
Self-care	Prevalence (%, 95% CI)	20.3 (11.3–33.6)	10.4 (5.9–17.6)	12.1 (3.6–33.3)	0.1818
Usual activities	Prevalence (%, 95% CI)	35.1 (23.7–48.5)	20.5 (13.3–30.2)	17.7 (6.1–41.5)	0.0888
Pain/discomfort	Prevalence (%, 95% CI)	45.6 (33.9–57.9)	41.7 (30.7–53.6)	29.6 (12.3–55.8)	0.5458
Anxiety/depression	Prevalence (%, 95% CI)	16.4 (9.6–26.7)	10.9 (5.5–20.2)	4.1 (0.7–20.7)	0.2469

Adjusted for age and sex

## Discussion

In this study, we investigated the prevalence of cardiovascular health indicators among stroke survivors in Korea between 2013 and 2018, and evaluated the changes in these indicators across 2013–2018.

A previous UK study looking for factors influencing the quality of life of stroke survivors reveals that diabetes and ischemic heart disease were significant factors [27]. Another study of Malaysians shows that obesity, smoking status, malnutrition risk, and physical activity level had an effect on the physical dimensions of the quality of life [28]. This study showed the relationship between overall cardiovascular health and quality of life in stroke survivors from the nationally representative cross-sectional survey.

With respect to health behaviors, the prevalence of smoking cessation was well maintained at > 80% across all three periods, whereas physical activity and healthy eating scores were < 10% in ideal standard groups. However, physical activity and healthy eating may be restricted depending on the degree of stroke sequelae. Across various health factors, the group with a total cholesterol level of < 200 mg/dL was relatively well managed during the three periods. However, the incidence of elevated blood pressure and fasting blood glucose was higher in the 0-point and 1-point groups than in the ideal group. In the total cholesterol levels of the 1-point group, there was a significant reduction in 2017–2018 compared to that in 2013–2014. However, other factors did not differ significantly across the three periods. As a result, the ideal cardiovascular health score group comprised 7.72%–8.14% of the participants during the survey period. A history of cardiovascular disease, such as stroke, is a strong risk factor for the onset of secondary cardiovascular disease, such as myocardial infarction, the identification and modification of active underlying vascular risk factors and life management are required [29–31].

In general, HRQoL declines after a stroke [14, 32], and cardiovascular health status and HRQoL have a complementary effect [33–35]. This is proved by the EQ-5D index score in the poor, intermediate, and ideal groups across all three periods in our study. HRQoL can lead to and affect the ability or willingness to maintain optimal cardiovascular health, and an optimal cardiovascular health status can lead to better HRQoL. In particular, strict management of physical activity and diet was low in this survey. In previous studies, physical activity [36–38] and a healthy diet [39] improved the HRQoL among stroke survivors. HRQoL can be improved by maintaining optimal physical activity levels and a healthy diet.

When analyzing the EQ-5D index score by period, stroke survivors who had mobility problems showed significant differences in poor, intermediate, and ideal cardiovascular health scores in 2015–2016 and 2017–2018. Stroke survivors have outcomes ranging from complete recovery to severe disability. Functional deficits that manifest as stroke sequelae may include cognitive, speech, sensory, and motor impairments; however, the most recognized deficit is motor impairment, which negatively impacts patients’ mobility and QoL [40, 41]. The results of our study, which shows that mobility was significantly different among the three groups classified by cardiovascular health scores, indicate that mobility is closely related to cardiovascular health management. Patients who have limited mobility due to stroke sequelae can be more vulnerable to problems with cardiovascular health management.

This study has several limitations. First, as secondary data were used, the sample size was small and the samples themselves differed across the various time periods. Second, unadjusted socioeconomic factors, other physical factors, and stroke severity might have affected the outcome. Third, it was difficult to analyze the cause of significant differences across the three periods due to the limitations imposed by cross-sectional studies. However, this study was valuable in that the relationship between cardiovascular health status and HRQoL among stroke survivors in Korea was calculated using the EQ-5D index score. The reproducibility of the results can be enhanced by observing the three periods. Further, the results of this study could be helpful in establishing policies for cardiovascular health management of stroke survivors in Korea.

## Conclusion

Stroke patients with high cardiovascular health status had higher HRQoL. This relationship between HRQoL and cardiovascular health among stroke patients was reconfirmed in this study. Our data will be useful as a clinical basis for establishing patient management strategies appropriate for this population. In the future, the mechanisms and interventions that facilitate optimal care among stroke survivors should be investigated.

## Data Availability

The datasets generated during and/or analyzed during the current study are available in the KNHANES repository (https://knhanes.cdc.go.kr/knhanes/sub03/sub03_02_05.do).

## Abbreviations

AHA: American heart association
BMI: Body mass index
HRQoL: Health-related quality of life
KHEI: Korean healthy eating index
KNHANES: Korea national health and nutrition examination survey
QoL: Quality of life
SE: Standard error

## References

[1] World Health Organization (The top 10 causes of death. Vol. 2020) Available from: https://www.who.int/news-room/fact-sheets/detail/the-top-10-causes-of-death. Accessed 29 Oct 2020.

[2] Cha YJ (2018). The economic burden of stroke based on South Korea’s national health insurance claims database. Int J Health Policy Manag.

[3] Feigin VL, Forouzanfar MH, Krishnamurthi R, Mensah GA, Connor M, Bennett DA (2014). Global and regional burden of stroke during 1990–2010: findings from the Global burden of disease study 2010. Lancet.

[4] Chin YY, Sakinah H, Aryati A, Hassan BM (2018). Prevalence, risk factors and secondary prevention of stroke recurrence in eight countries from south, east and Southeast Asia: a scoping review. Med J Malaysia.

[5] Sandeep K, Magdy HS, Louis RC (2010). Medical complications after stroke. Lancet Neurol.

[6] Hardie K, Hankey GJ, Jamrozik K, Broadhurst RJ, Anderson C (2004). Ten-year risk of first recurrent stroke and disability after first-ever stroke in the Perth community stroke study. Stroke.

[7] Hankey GJ (2014). Secondary stroke prevention. Lancet Neurol.

[8] Pendlebury ST, Rothwell PM (2009). Risk of recurrent stroke, other vascular events and dementia after transient ischaemic attack and stroke. Cerebrovasc Dis.

[9] Esenwa C, Gutierrez J (2015). Secondary stroke prevention: challenges and solutions. Vasc Health Risk Manag.

[10] Guzik A, Bushnell C (2017). Stroke epidemiology and risk factor management. Continuum (Minneap Minn).

[11] Boehme AK, Esenwa C, Elkind MS (2017). Stroke risk factors, genetics, and prevention. Circ Res.

[12] Caprio FZ, Sorond FA (2019). Cerebrovascular disease: primary and secondary stroke prevention. Med Clin North Am.

[13] De Wit L, Theuns P, Dejaeger E, Devos S, Gantenbein AR, Kerckhofs E (2017). Long-term impact of stroke on patients’ health-related quality of life. Disabil Rehabil.

[14] Yeoh YS, Koh GC, Tan CS, Tu TM, Singh R, Chang HM (2019). Health-related quality of life loss associated with first-time stroke. PLoS One.

[15] van Mierlo M, van Heugten C, Post MWM, Hoekstra T, Visser-Meily A (2018). Trajectories of health-related quality of life after stroke: results from a one-year prospective cohort study. Disabil Rehabil.

[16] Jeon NE, Kwon KM, Kim YH, Lee JS (2017). The factors associated with health-related quality of life in stroke survivors age 40 and older. Ann Rehabil Med.

[17] Kwon SY, Park JH, Kim WS, Han K, Lee Y, Paik NJ (2018). Health-related quality of life and related factors in stroke survivors: data from Korea National Health and nutrition examination survey (KNHANES) 2008 to 2014. PLoS ONE.

[18] Kweon S, Kim Y, Jang MJ, Kim Y, Kim K, Choi S (2014). Data resource profile: the Korea National health and nutrition examination survey (KNHANES). Int J Epidemiol.

[19] Eun MY, Seo WK, Lee J, Kim M, Kim J, Kim JH (2013). Age-dependent predictors for recurrent stroke: the paradoxical role of triglycerides. Eur Neurol.

[20] Lloyd-Jones DM, Hong Y, Labarthe D, Mozaffarian D, Appel LJ, Van Horn L (2010). Defining and setting national goals for cardiovascular health promotion and disease reduction: the American Heart Association’s strategic impact goal through 2020 and beyond. Circulation.

[21] Allen NB, Badon S, Greenlund KJ, Huffman M, Hong Y, Lloyd-Jones DM (2015). The association between cardiovascular health and health-related quality of life and health status measures among U.S. adults: a cross-sectional study of the National health and nutrition examination surveys, 2001–2010. Health Qual Life Outcomes.

[22] WHO Expert Consultation (2004). Appropriate body-mass index for Asian populations and its implications for policy and intervention strategies. Lancet.

[23] IPAQ (2005) Guidelines for data processing and analysis of the international physical activity questionnaire. https://www.researchgate.net/file.PostFileLoader.html?id=5641f4c36143250eac8b45b7&assetKey=AS%3A294237418606593%401447163075131. Accessed 29 Oct 2020

[24] Shim JS, Jung SJ, Kim HC (2019). Self-reported diet management, dietary quality, and blood pressure control in Korean adults with hypertension. Clin Hypertens.

[25] Yook SM, Park S, Moon HK, Kim K, Shim JE, Hwang JY (2015). Development of Korean healthy eating index for adults using the Korea National health and nutrition examination survey data. J Nutr Health.

[26] Lee YK, Nam HS, Chuang LH, Kim KY, Yang HK, Kwon IS (2009). South Korean time trade-off values for EQ-5D health states: modeling with observed values for 101 health states. Value Health.

[27] Patel MD, McKevitt C, Lawrence E, Rudd AG, Wolfe CDA (2007). Clinical determinants of long-term quality of life after stroke. Age Ageing.

[28] Wong HJ, Lua PL, Harith S, Ibrahim KA (2021). Health-related quality of life profiles and their dimension-specific associated factors among Malaysian stroke survivors: a cross sectional study. Health Qual Life Outcomes.

[29] Qureshi AI, Suri MF, Guterman LR, Watzke RC, Francis PJ (2001). Ineffective secondary prevention in survivors of cardiovascular events in the US population: report from the Third National health and nutrition examination survey. Arch Intern Med.

[30] Brenner DA, Zweifler RM, Gomez CR, Kissela BM, Levine D, Howard G (2010). Awareness, treatment, and control of vascular risk factors among stroke survivors. J Stroke Cerebrovasc Dis.

[31] Prabhakaran S, Chong JY (2014). Risk factor management for stroke prevention. Continuum (Minneap Minn).

[32] Labberton AS, Augestad LA, Thommessen B, Barra M (2020). The association of stroke severity with health-related quality of life in survivors of acute cerebrovascular disease and their informal caregivers during the first year post stroke: a survey study. Qual Life Res.

[33] Singh RJ, Chen S, Ganesh A, Hill MD (2018). Long-term neurological, vascular, and mortality outcomes after stroke. Int J Stroke.

[34] Myint PK, Surtees PG, Wainwright NW, Luben RN, Welch AA, Bingham SA (2007). Physical health-related quality of life predicts stroke in the EPIC-Norfolk. Neurology.

[35] Pinheiro LC, Reshetnyak E, Sterling MR, Richman JS, Kern LM, Safford MM (2019). Using health-related quality of life to predict cardiovascular disease events. Qual Life Res.

[36] Rand D, Eng JJ, Tang PF, Hung C, Jeng JS (2010). Daily physical activity and its contribution to the health-related quality of life of ambulatory individuals with chronic stroke. Health Qual Life Outcomes.

[37] Cook P, Sunnerhagen KS, Persson HC (2020). Level of physical activity is positively correlated with perceived impact on life 12 months after stroke: a cross-sectional study. J Rehabil Med.

[38] Jeon NE, Kwon KM, Kim YH, Lee JS (2017). The factors associated with health-related quality of life in stroke survivors age 40 and older. Ann Rehabil Med.

[39] Chiba R, Tominaga S, Mikami K, Kitajima M, Urushizaka M, Tomisawa T (2019). Factors influencing quality of life in stroke patients: Focus on eating habits. J Stroke Cerebrovasc Dis.

[40] Alawieh A, Zhao J, Feng W (2018). Factors affecting post-stroke motor recovery: implications on neurotherapy after brain injury. Behav Brain Res.

[41] Langhorne P, Coupar F, Pollock A (2009). Motor recovery after stroke: a systematic review. Lancet Neurol.

